# Validation of self-reported human papillomavirus vaccination in young adult men who have sex with men

**DOI:** 10.1080/21645515.2024.2371179

**Published:** 2024-07-07

**Authors:** Eric P.F. Chow, Christopher K. Fairley, Sidney Atkinson, Catriona S Bradshaw, Marcus Y. Chen

**Affiliations:** a Melbourne Sexual Health Centre, Alfred Health, Melbourne, Australia; b School of Translational Medicine, Faculty of Medicine, Nursing and Health Sciences, Monash University, Melbourne, Australia; c Centre for Epidemiology and Biostatistics, Melbourne School of Population and Global Health, The University of Melbourne, Melbourne, Australia

**Keywords:** HPV, vaccine, accuracy, prevention, gay, prevention, cancer

## Abstract

The Victorian Government introduced a time-limited human papillomavirus (HPV) catch-up program for gay, bisexual, and other men who have sex with men (GBMSM) aged ≤ 26 years in 2017–2019. We conducted a retrospective observational study to examine the accuracy of the self-report of HPV vaccination status using computer-assisted self-interviewing versus their immunization history via electronic health records. We included GBMSM aged 23–30 years visiting the Melbourne Sexual Health Centre (MSHC) in 2020–2021 because they were age-eligible for the HPV catch-up program in Victoria, Australia. Individuals who were unsure about their vaccination status were categorized as ‘unvaccinated’. Of the 1,786 eligible men, 1,665 men self-reported their HPV vaccination status: 48.8% (*n* = 812) vaccinated, 17.4% (*n* = 289) unvaccinated, and 33.9% (*n* = 564) unsure. Self-reported HPV vaccination had a sensitivity of 61.3% (95%CI: 58.3 to 64.2%; 661/1079), a specificity of 74.2% (95%CI: 70.5 to 77.7%; 435/586), a positive predictive value of 81.4% (95%CI: 78.6 to 84.0%; 661/812), a negative predictive value of 51.0% (95%CI: 47.6 to 54.4%; 435/853), and an accuracy of 52.6% (95%CI: 50.1 to 55.0%). Our results showed that only half of GBMSM know and report their HPV vaccination status correctly. Novel approaches such as digital vaccine passports may be useful for individuals to accurately report their vaccination status to guide accurate clinical decisions and management.

## Introduction

As of 2022, 130 out of 193 World Health Organization (WHO) Member States have introduced the human papillomavirus (HPV) vaccine on their national routine immunization schedule,^1^ and numerous epidemiological studies have reported significant reductions in genital warts, HPV infection, pre-cancers and cervical cancers in females after the introduction of the HPV vaccination program.^2–5^ Studies have also shown that female-only HPV vaccination programs not only benefit females but also unvaccinated heterosexual males due to herd protection.^3–8^ However, the same magnitude of herd protection among gay, bisexual, and other men who have sex with men (GBMSM) is not observed due to their sexual practices.^9^ GBMSM are at higher risk of acquiring HPV and anal cancer;^10–12^ and thus, vaccinating GBMSM is important in protecting them against HPV and HPV-associated cancers. In Australia, some states including Victoria, introduced HPV vaccination catch-up programs for GBMSM; however, these programs had limited timeframes and were not ongoing.^13^

Many countries (60 WHO Member States as of January 2024) have now introduced gender-neutral HPV vaccination programs to promote gender equity and reduce the burden of HPV.^14^ Accurately reporting HPV vaccination status is important so that unvaccinated individuals can be identified and receive vaccination. Some countries, including Australia, have established immunization registries to better capture vaccination status and track population coverage. The Australian Immunization Register (AIR) is a national register that records vaccines given within the free national immunization program to people of all ages in Australia. It is a mandatory requirement for vaccination providers to report all vaccinations funded by the National Immunization Program (since 1 July 2021).^15^ However, vaccination providers are encouraged (but it is not mandatory) to report vaccines that are not funded by the National Immunization Program. This included HPV vaccinations administered through a time-limited catch-up program for GBMSM.

There have been very limited studies examining the accuracy of self-reported HPV vaccination status among GBMSM.^16^ A previous Australian study has examined the accuracy of self-reported HPV vaccination status compared with the immunization registry among adolescent GBMSM who were eligible for the school-based HPV vaccination program funded by the National Immunization Program.^17^ The primary aim of this study was to examine the accuracy of self-reported HPV vaccination status compared to electronic health records among young adult GBMSM eligible for the time-limited catch-up program and those who missed the school-based HPV vaccination program. The secondary aim was to examine whether certain characteristics (e.g. HIV status, use of HIV pre-exposure prophylaxis (PrEP)) would affect the sensitivity and specificity of self-reported HPV vaccination status.

## Methods

### Study design and population

This was a retrospective cross-sectional study among GBMSM attending the Melbourne Sexual Health Centre, Victoria, Australia. In this analysis, we included GBMSM who aged 23–29 years in 2020 or aged 24–30 years in 2021, and attended MSHC at least once in 2017–2019. MSHC remained open in 2020–2021 during the COVID-19 pandemic.^18^ GBMSM was defined as men who had had sex with another man in the last 12 months. This project was approved by the Alfred Hospital Ethics Committee, Melbourne, Australia (681/21). Informed consent was waived because of the retrospective nature of the study.

**Figure 1.**
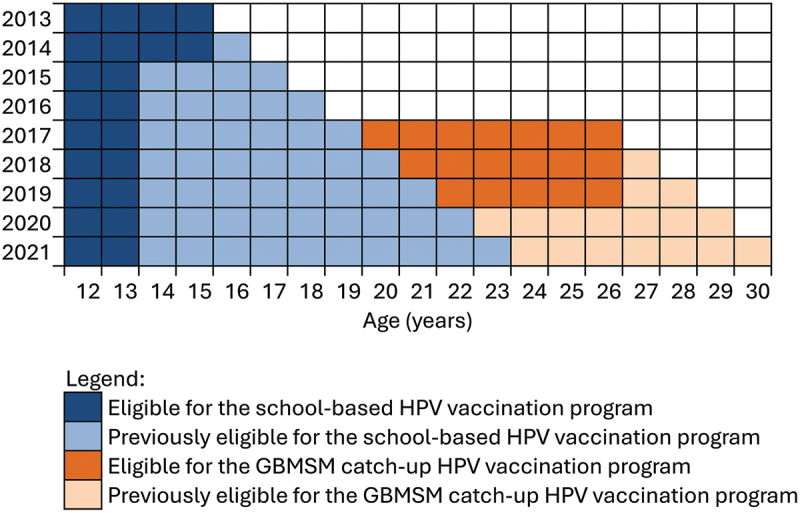
Age cohort for the human papillomavirus vaccination programme for male individuals in Victoria, Australia.

Figure 1 illustrates the age cohort for the national gender-neutral school-based HPV vaccination program and the Victorian time-limited HPV vaccination program for GBMSM. In brief, the gender-neutral school-based HPV vaccination program started in 2013, and boys aged 12–15 years were eligible to receive three doses of the quadrivalent HPV vaccines free of charge. As of 2020, males aged up to 22 years would have been offered the HPV vaccine through the national school-based HPV vaccination program. Separately, the Victorian government introduced a time-limited HPV vaccination program for GBMSM aged ≤26 years in 2017–2019.^13,19^ Eligible GBMSM were able to receive three doses of the quadrivalent HPV vaccines free of charge at sexual health clinics or immunization providers. Males aged 23–29 years in 2020 or males aged 24–30 years in 2021 would have been eligible for the HPV vaccine through the time-limited HPV vaccination program but not eligible for the national gender-neutral school-based HPV vaccination program; thus, this age cohort was selected for this analysis. Males who were eligible for the school-based HPV vaccination program were not eligible in this analysis because (1) separate consent would be required to verify their vaccination records through the AIR as they would have received the vaccine at school instead of at our clinic, and this could not be done from a retrospective data analysis, and (2) the accuracy of self-reported HPV vaccine using immunization registry data among adolescent GBMSM was examined in a previous study.^17^

### Data collection

Upon arrival at the clinic, new clients and existing clients who have not attended MSHC within three months are asked to complete a questionnaire via computer-assisted self-interview (CASI). This is a questionnaire that collects clients’ demographic characteristics and sexual history for routine standard care and management at MSHC. In this questionnaire, clients are also asked whether they have been vaccinated against HPV. Clients could choose ‘yes,’ ‘no’ or ‘unsure’ if they were not sure about their vaccination status. For clients with multiple visits at MSHC, we only included the data from their first visit during the study period. A chart review was performed to verify clients’ immunization history via electronic health records at MSHC by collecting data on the number of HPV vaccine doses administered and the date of the last dose.

### Statistical analyses

We calculated the sensitivity, specificity, positive predictive value, negative predictive value, positive likelihood ratio, negative likelihood ratio, and accuracy for self-reported HPV vaccination status via CASI and used electronic medical records at MSHC as the reference standard among all eligible individuals. Individuals who were unsure about their vaccination status were considered unvaccinated when calculating the diagnostic accuracy parameters. Sensitivity is the proportion of men who correctly reported being vaccinated among all vaccinated men verified electronic health records. Specificity is the proportion of men who reported being unvaccinated or were unsure among all unvaccinated men verified against electronic health records. Positive predictive value is the proportion of men who correctly reported being vaccinated among all those who self-reported being vaccinated. Negative predictive value is the proportion of men who correctly reported being unvaccinated or unsure among all those who self-reported being unvaccinated or unsure. Positive likelihood ratio is defined as the probability of men who self-reported being vaccinated among those who were vaccinated divided by the probability of men who self-reported being vaccinated among those who were unvaccinated. The negative likelihood ratio is the probability of men who self-reported being unvaccinated or unsure among those who were vaccinated divided by the probability of individuals who self-reported being unvaccinated or unsure among those who were unvaccinated. The accuracy was defined as the total of individuals who correctly self-reported as vaccinated or unvaccinated divided by the total number of individuals. We repeated the analyses by excluding individuals who were unsure about their vaccination status. We calculated the kappa statistics to determine the agreement between self-reported vaccination status and registry records. The level of agreement was categorized based on the κ statistics as none (0–0.20), minimal (0.21–0.39), weak (0.40–0.59), moderate (0.60–0.79), strong (0.80–0.90), and almost perfect (>0.90).^20^ We stratified men into different groups based on their characteristics (e.g. age, residency, time since the last HPV vaccine, and the number of HPV doses, HIV status, PrEP use and number of male partners in the last 3 months) and performed additional analyses by calculating the sensitivity and specificity in each subgroup. The 95% confidence intervals (CI) of the point estimates were calculated using binomial exact methods. All statistical analyses were performed in Stata (version 17; StataCorp LLC).

## Results

**Figure 2.**
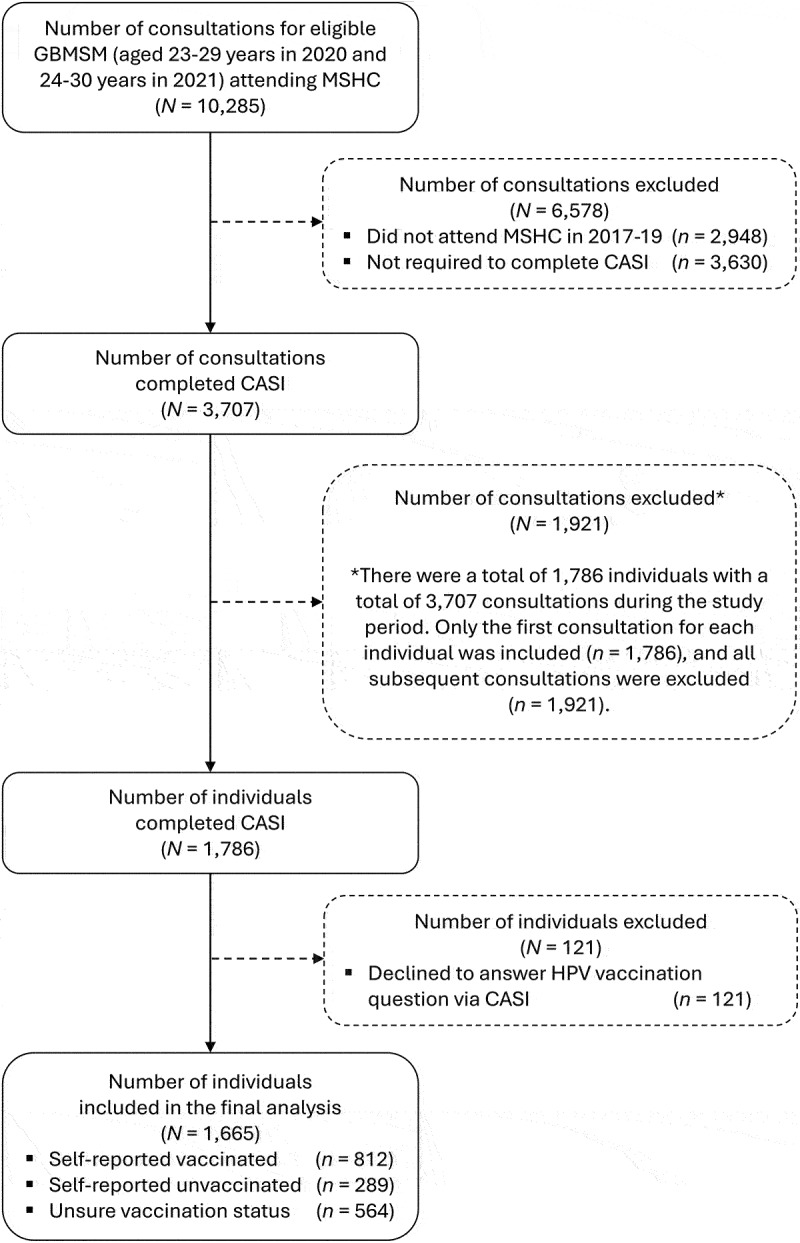
Flow chart of study participant selection.

There was a total of 10,285 consultations for GBMSM aged 23–29 years in 2020 and 24–30 years in 2021 attending MSHC. We excluded 6,578 consultations because 2,948 consultations were individuals who did not attend MSHC between April 2017 and October 2019, and 3,630 consultations where the individuals were not required to complete CASI. The remaining 3,707 consultations among 1,786 individual GBMSM met the inclusion criteria (Figure 2). Of the 1,786 individual GBMSM, 121 (6.8%) declined to answer the HPV vaccination question and were excluded. The final analysis included 1,665 individual GBMSM.

Of the 1,665 GBMSM included in the final analysis, the median age was 27 (IQR 25 to 28) years. Half (49.9%, *n* = 831) were born in Australia, 48% (*n* = 804) were born outside Australia, and 1.8% (*n* = 30) declined to report their country of birth. The median number of male partners in the last 12 months was 5 (IQR 2 to 10), and 9.8% (*n* = 163) also had a female partner in the last 12 months. There were 48.8% (*n* = 812) of men who self-reported being vaccinated against HPV, 17.4% (*n* = 289) reported being unvaccinated, and 33.9% (*n* = 564) were unsure about whether they had been vaccinated.

All 1,665 GBMSM had attended MSHC at least once between April 2017 and October 2019 during the roll-out of the time-limited HPV vaccination program for GBMSM in Victoria; thus, their vaccination status was verified through the electronic health record at MSHC. There were 64.8% (1,079/1,665) of men had received at least one dose of HPV vaccine administered at MSHC and were verified on the MSHC’s electronic health record (Table 1), and these men were categorized as vaccinated. Of the 1,079 men who had received at least one dose of HPV vaccine, the median time since their last dose was 1.7 (IQR 1.1 to 2.3) years. Most men received three doses of HPV vaccines (63.4%, 684/1079), 17.8% (192/1079) received two doses and 18.8% (203/1079) received one dose.

**Table 1.** Confusion matrix and corresponding likelihood ratios comparing self-reported vaccination status using computer-assisted self-interviewing with electronic health records among 1,665 GBMSM attending the Melbourne Sexual Health Centre.

	Electronic health records			
Self-reported vaccination status	Vaccinated	Unvaccinated	Total	Likelihood ratio (95% CI)
Yes	661	151	812	2.4 (2.1 to 2.7)
No	75	214	289	0.19 (0.15 to 0.24)
Unsure	343	221	564	0.84 (0.74 to 0.97)
Total	1079	586	1665	

There were 82 men who did not have any HPV vaccination records on MSHC’s electronic health record but had information written by the clinician in their medical notes indicating they had been vaccinated, this included seven who had had the vaccine outside Australia, 14 reported they had had the vaccine at school, and 24 had had the vaccine at other healthcare services in Australia (e.g. GP); and these men were categorized as unvaccinated in the electronic health records because these were self-reported without any official documents verifying their vaccination history.

There were 25.1% (418/1665) of men who self-reported being unvaccinated or unsure but were vaccinated as per their electronic health record (Table 1). Similarly, 9.1% (151/1665) of men self-reported being vaccinated but their electronic health records showed they had not. Overall, 875 men correctly self-reported as vaccinated or unvaccinated, and the accuracy of self-reported HPV vaccination status was 52.6% (95%CI: 50.1% to 55.0%). Of the men who were vaccinated, 61.3% (661/1079) correctly self-reported being vaccinated (sensitivity = 61.3%; 95%CI: 58.3% to 64.2%) (Table 2). When categorizing men who were unsure about their vaccination status as unvaccinated, 74.2% (435/586) of men correctly self-reported being unvaccinated or unsure of their vaccination status (specificity = 74.2%, 95%CI: 70.5% to 77.7%). The positive likelihood ratio was 2.4 (95%CI: 2.1 to 2.7), and the negative likelihood ratio was 0.52 (95%CI: 0.48 to 0.57). The Kappa coefficient was (κ = 0.321; 95%CI: 0.278 to 0.364) suggesting a fair agreement between self-reported vaccination status and electronic health records. We also performed additional analyses by excluding men who were unsure about their vaccination status, the sensitivity increased to 89.8% (88.0% to 91.6%) but the specificity decreased to 58.6% (55.7% to 61.5%) (Table 2).

**Table 2.** Sensitivity, specificity, predictive values and likelihood ratios of self-reported HPV vaccination status among GBMSM using electronic health records as the reference standard.

Parameters	All men (*N*=1665), Measure (95% CI)	All men excluding those who were unsure about their vaccination status (*N*=1101), Measure (95% CI)
Sensitivity	61.3% (58.3 to 64.2%)	89.8% (88.0 to 91.6%)
Specificity	74.2% (70.5 to 77.7%)	58.6% (55.7 to 61.5%)
Positive predictive value	81.4% (78.6 to 84.0%)	81.4% (79.1 to 83.7%)
Negative predictive value	51.0% (47.6 to 54.4%)	74.1% (71.5 to 76.6%)
Positive likelihood ratio	2.4 (2.1 to 2.7)	2.2 (1.9 to 2.5)
Negative likelihood ratio	0.52 (0.48 to 0.57)	0.17 (0.14 to 0.22)
Kappa coefficient	0.32 (0.28 to 0.36)	0.51 (0.46 to 0.57)
Accuracy	52.6% (50.1 to 55.0%)	79.5% (77.0 to 81.8%)

CI: Confidence intervals.

The sensitivity of self-reported vaccination status was significantly higher among those who had 3 doses (70.6%; 95%CI: 68.1% to 73.1%) followed by 2 doses (49.0%; 95%CI: 41.7% to 56.3%) and 1 dose (41.4%; 95% CI: 34.5% to 48.5%) (*p* < .001) (Table 3). PrEP users had a higher sensitivity (70.0%; 95%CI: 66.2% to 73.8%) of self-reported vaccination status compared to HIV-negative GBMSM (57.0%; 95%CI: 54.0% to 60.0%) and people living with HIV (49.2%; 95%CI: 38.4% to 59.9%) (*p* < .001). However, sensitivity did not differ in age (*p* = .404), residency (*p* = .762), time since the last HPV dose (*p* = .719), and the number of male partners in the last 12 months (*p* = .081). The specificity of self-reported vaccination status was similar across different characteristics.

**Table 3.** Sensitivity and specificity of self-reported HPV vaccination status using computer-assisted self-interviewing compared to electronic health records among 1,665 GBMSM, stratified by different characteristics.

	Electronic health records		Self-reported vaccination status			Sensitivity	Specificity
Characteristics	Vaccinated	Unvaccinated	Yes	No	Unsure	Measure (95% CI)	Measure (95% CI)
Overall	1079	586	661	75	343	61.3% (58.3 to 64.2%)	74.2% (70.5 to 77.7%)
Age group							
*23–26*	518	247	324	29	165	62.5% (59.1 to 66.0%)	70.5% (67.2 to 73.7%)
*27–30*	561	339	337	46	178	60.1% (56.9 to 63.3%)	77.0% (74.2 to 79.7%)
Residency							
*Australian-born citizens*	535	296	328	25	182	61.3% (58.0 to 64.6%)	71.6% (68.6 to 74.7%)
*Overseas-born residents*^*a*^	277	130	164	23	90	59.2% (54.4 to 64.0%)	74.6% (70.4 to 78.8%)
*Overseas-born recent arrivals*^*b*^	246	151	153	27	66	62.2% (57.4 to 67.0%)	78.8% (74.8 to 82.8%)
Time since the last documented HPV vaccine dose							
*<1 year ago*	217	586	130	23	64	59.9% (53.6 to 66.5%)	74.2% (71.2 to 77.3%)
*1 year ago*	425	586	257	25	143	60.5% (55.6 to 65.1%)	74.2% (71.5 to 76.9%)
*≥2 years ago*	437	586	274	27	136	62.7% (58.0 to 67.2%)	74.2% (71.6 to 76.9%)
Documented the number of HPV vaccine doses							
*One*	203	586	84	32	87	41.4% (34.5 to 48.5%)	74.2% (71.2 to 77.3%)
*Two*	192	586	94	14	84	49.0% (41.7 to 56.3%)	74.2% (71.2 to 77.3%)
*Three*	684	586	483	29	172	70.6% (68.1 to 73.1%)	74.2% (71.8 to 76.6%)
HIV status & PrEP use							
*HIV-negative*	630	395	359	56	215	57.0% (54.0 to 60.0%)	79.2% (76.8 to 81.7%)
*PrEP users*	390	167	273	16	101	70.0% (66.2 to 73.8%)	64.1% (60.1 to 68.1%)
*People living with HIV*	59	24	29	3	27	49.2% (38.4 to 59.9%)	62.5% (52.1 to 72.9%)
Number of male partners in the last 12 months							
*≤5*	620	359	366	52	202	59.0% (55.9 to 62.1%)	80.8% (78.3 to 83.3%)
*>5*	459	227	295	23	141	64.3% (60.7 to 67.9%)	63.9% (60.3 to 67.5%)

^a^Overseas born individuals who have resided in Australia for more than four years.

^b^Overseas born individuals who have resided in Australia for less than four years.

## Discussion

This cross-sectional study examines the accuracy of self-reported HPV vaccination using CASI compared to clinic’s electronic medical records among young GBMSM aged 23–30 years attending a sexual health service. Our data suggest that only 53% of GBMSM actually knew their correct HPV vaccination status. The poor sensitivity of 61% suggests that self-reported vaccination can only identify less than two-thirds of vaccinated GBMSM. Although we found that the sensitivity of self-reported vaccination status has greatly improved to 90% after excluding men who were unsure about their vaccination status, this is not practical in clinical settings.

To our knowledge, there have been only three studies examining the accuracy of self-reported HPV vaccination status among men, and our study has the largest sample size compared to the previous three studies. However, direct comparisons with previous studies are difficult due to the different measurements and indicators used. The first study was published by Thomas et al. (2018) among 400 young men aged 13 to 26 years in the United States.^21^ The authors found that the accuracy of self-reported vaccination status was 80%, which was higher than our estimate (53%); however, most men in Thomas et al.’s study were heterosexuals with only 9% of men who had a male partner in the last 3 months. Furthermore, the authors asked the participants to report their vaccination status as either vaccinated or not vaccinated, and there was no option of unsure. After excluding those who were unsure about their vaccination status, the accuracy of self-reported vaccination in our study was 80%, which is consistent with Thomas et al.’s study. The authors did not report other diagnostic parameters such as sensitivity and specificity.

The second study was published by Forward et al. (2022) among 751 GBMSM and transgender women aged 18 to 26 years in the United States.^22^ The authors reported a sensitivity of 83% which was higher than the estimate in our study (61%). The higher sensitivity is likely attributed to the inclusion of both clinic electronic medical records and statewide immunization information systems in the US study, and this is also suggested in other vaccinations such as influenza vaccination.^23^ Furthermore, our findings are also consistent with Forward et al.’s study showing that those who have received ≥ 3 doses have the highest sensitivity compared to those who have received 1 or 2 doses.^22^ It has been shown that self-reporting the number of doses is less accurate than any doses of HPV vaccine.^22^ However, the number of doses is important for clinical decisions if multi-dose vaccination is recommended (e.g. people with immunocompromising conditions). The authors only reported sensitivity but no other parameters such as specificity and predictive values due to the limitations of the health records and registry data.

The third study was published by Chow et al. (2021) among 192 GBMSM aged 16 to 20 years in Australia.^17^ We found that the sensitivity of self-reported vaccination status was higher in the current study (61%) compared to our previous study (48%) although the current study used in-house electronic health records as the reference standard while the previous study used documented vaccination records on the HPV or Immunization registries.^17^ Different age cohorts may also explain the difference in sensitivity as past studies have found that the sensitivity of self-reported vaccination status improves with increasing age.^22^ However, both studies reported similar positive and negative likelihood ratios, suggesting self-reported vaccination status is not useful for ruling in being vaccinated as both positive likelihood ratios were less than 10; and it is also not useful for ruling out being vaccinated as the negative likelihood ratios were greater than 0.1.

Electronic medical records could be considered as the gold standard to validate self-reported vaccination status, particularly when settings do not have a vaccine registry. A study conducted by Rolnick et al. (2013) in the United States examined the accuracy of self-reported vaccination status compared to electronic medical records on eight vaccines, and they found that the sensitivity and specificity vary by vaccines but not by demographic characteristics such as age and ethnicity.^24^ For example, the sensitivity ranges from 62.5% for hepatitis A to 92.1% for tetanus; while the specificity ranges from 11% for tetanus to 90.7% for pneumococcal.^24^ The variations in self-reporting vaccination may be due to several factors such as the time of vaccination (i.e. where individuals received their vaccination during childhood or adulthood), the purpose of vaccination (i.e. travel, employment, outbreak), and the number of doses or boosters of the vaccine. However, it is important to note that in-house electronic medical records may not be reliable and accurate if the individuals receive the vaccines outside the health system or service.

The major strength of this study is that this is the first time the sensitivity of self-reported vaccination status has been examined, stratified by HIV status and PrEP use. We found that PrEP users are more likely to correctly report their vaccination status compared to non-PrEP users and people living with HIV. This may be due to PrEP users having a higher sexual health literacy and proactively taking care of their sexual wellbeing including a higher vaccination coverage in other vaccines such as mpox.^25,26^ The large sample size of 1,665 GBMSM is another strength as it provides a more precise estimate compared to the previous three studies.^17,21,22^ However, our study also has several limitations. First, potential misclassification of true vaccination status might have occurred because we were only able to link the electronic health records at our clinic. During the time-limited HPV vaccination program in Victoria, free vaccines were also offered not only through sexual health clinics but also through other immunization providers. Some men may have received their HPV vaccination elsewhere. There were 82 men self-reported they had received HPV vaccines outside MSHC but we were unable to verify this. Furthermore, other free time-limited vaccinations including hepatitis A and meningococcal ACWY were also available in Victoria at the same time as the time-limited HPV vaccination program for GBMSM.^27,28^ It is possible that some men might be confused with other vaccinations. Self-reported vaccination status could be subject to false negatives where vaccinated individuals are incorrectly classified as unvaccinated and getting an unnecessary vaccine, and false positives where unvaccinated individuals are incorrectly classified as vaccinated and missing a vaccine. Second, we did not obtain consent from clients to verify the vaccination status on AIR due to the nature of retrospective data analysis. It is not a mandatory requirement to report vaccines if the individuals receive the vaccine through a time-limited catch-up program; and thus, accessing AIR data might not be helpful. Third, selection bias might have occurred due to our very restrictive inclusion criteria to ensure men were age-eligible for the time-limited HPV vaccination program. Therefore, our data may not be generalizable to all GBMSM, particularly to those who are not in this age group.

To conclude, the low sensitivity of self-reported HPV vaccination status means patient history has limited accuracy for verifying past HPV vaccination, and caution must be taken when using self-reported vaccination data while other verification methods are not available or accessible. Almost half of the men did not accurately self-report their HPV vaccination status, particularly one-third of men were unsure about their vaccination status. Accurately reporting HPV vaccination status is important for clinical decisions and management and for monitoring vaccination coverage to evaluate the impact of vaccination programs. In March 2021, MSHC introduced a free service to clients where they can receive an e-mail summary of their clinical visit to the service (including vaccinations they had received on the day of the visit),^29^ this additional vaccination record could provide the ability for the clients to report their vaccination status outside MSHC. Novel approaches such as digital vaccine passports may be useful for individuals to track their immunization records.

## Data Availability

All data analyzed during this study are included in this article.

## Conference presentation

These data were presented as a poster presentation (#4811) at the EUROGIN (EUropean Research Organisation on Genital Infection and Neoplasia) 2023 in Bilbao, Spain, February 8–11, 2023.
